# Mechanical and electromagnetic shielding properties of carbon fabric with graphene nanoplatelets reinforced epoxy composites

**DOI:** 10.1038/s41598-025-00634-x

**Published:** 2025-05-06

**Authors:** R. Suresha, H. K. Sachidananda, B. Shivamurthy, N. Kumar Swamy, Sampath Parasuram

**Affiliations:** 1 School of Engineering & Information Technology, Department of Electrical & Electronics Engineering, Manipal Academy of Higher Education, Dubai, United Arab Emirates; 2 School of Engineering & Information Technology, Department of Mechanical Engineering, Manipal Academy of Higher Education, Dubai, United Arab Emirates; 3 Department of Mechanical & Industrial Engineering, Manipal Institute of Technology, Manipal Academy of Higher Education, Manipal, 576104 India; 4 Department of Chemistry, JSS Science & Technology University, Mysuru, India; 5 Department of Materials Engineering, Indian Institute of Science, Bangalore, India

**Keywords:** Electromagnetic shielding effectiveness, Carbon fabric, Epoxy, Tensile properties, Vickers hardness, Engineering, Materials science

## Abstract

In this work, carbon fabric-reinforced epoxy (CF/E) and carbon fabric/graphene nanoplatelets (1 wt%, 2 wt% and 3 wt%) reinforced epoxy (CF/GNP/E) laminates (one-layer, two-layer, and four-layer) were prepared using the hand layup method. Further, according to ASTM standards, the test samples were machined using abrasive waterjet machining, and tensile properties and hardness were studied. It was found that tensile strength modulus and strain-to-failure improved due to the addition of GNPs into the epoxy matrix in the composite; the CF/1GNP/E composite showed the highest tensile strength modulus and strain-to-failure. Further addition of GNPs of more than 2 wt% deteriorates the mechanical properties due to agglomeration. The electromagnetic interference shielding effectiveness by absorption and reflection of all the samples was investigated using the measured S-parameters in an X-band frequency range. The effectiveness of electromagnetic interference shielding increased with the addition of GNPs, and the multi-layer structure in the composites showed absorption-dominated electromagnetic shielding. The CF/3GNP/E 4-layer composite showed the highest (32.4dB) shielding effectiveness and was recommended for commercial application in the X-band frequency range.

## Introduction

The importance of electromagnetic interference (EMI) protection in modern aircraft is a significant area of research. Modern aircraft control increasingly depends on digital systems, electronic devices, and instruments. In addition, passengers also use various electronic gadgets, contributing to electromagnetic (EM) waves that can cause EMI. Due to the EMI effect, electronic devices malfunctioning and even damage to critical data and services onboard the aircraft were noticed^1^. Aircraft are also exposed to natural EM radiation due to thunderstorms, solar flares, and electrostatic discharges encountered during flight. These sources further elevate the risk of EMI, making aircraft particularly vulnerable to such threats.

In pursuing lighter and stronger materials, carbon fibre-reinforced epoxy (CF/E) composites have replaced mainly traditional aluminium and ferrous alloys in aircraft construction. CF/E composites offer numerous benefits, such as high strength, stiffness, low weight, high durability, thermal stability, and fatigue resistance. However, while these materials excel in structural integrity, their susceptibility to EMI is a growing concern^2^. Thus, in addition to their impressive mechanical properties, CF/E composites must have effective electromagnetic wave shielding capabilities, especially in the 8–12 GHz frequency range for aircraft applications. This frequency range is crucial for aircraft, radar, avionics, wireless communications, and computer networks^2^.

Based on the above, investigating the electromagnetic interference shielding effectiveness (EMI SE) of CF/E composites at X-band frequencies has become an important research area. Hence, the goal is to ensure that CF/E composites not only meet the structural needs of modern aircraft but also protect against the potential detriments of EMI SE, ensuring the safety, functionality, and longevity of both aircraft systems and onboard equipment. This research has broad implications for material engineering, as it seeks to improve both the safety and performance of aircraft while also contributing to the ongoing development of materials that can address the evolving challenges posed by electromagnetic threats in modern aviation.

As per the available literature, the EMI SE of carbon fibre-reinforced polymer (CFRP) composites is influenced by several key factors. (i) Direction of fibre orientation: The fibre orientation in CFRP composites plays a crucial role in determining the EMI SE. Unidirectional (UD) fibre-reinforced composites tend to have less EMI SE compared to bidirectional (BD), three-dimensional (3D) or multidirectional (MD) fibre-reinforced composites^3,25^. Hence, BD and 3D fabric-reinforced composites are better materials for aircraft because they have good EMI SE and structural properties. (ii) Layer architecture of reinforcements: The layering or stacking sequence of the reinforcement in CFRP composites also impacts EMI SE. The arrangement of the fibre layers, such as unidirectional, bidirectional, or other configurations, alters the distribution of electromagnetic waves and affects EMISE^4^. Also, the number of layers reinforced in the composites influences EMI SE. (iii) Type of reinforcement: The fibre used in the composite affects its overall performance in EMI SE. For instance, continuous fibre typically provides superior shielding performance compared to short fibre. UD carbon fabric, though effective, is generally outperformed by BD carbon fabric, which offers better balance in terms of mechanical properties, structural integrity, and EMI SE^3–6^&^7^. From the literature, it is found that various researchers investigated EMI SE of engineered carbon fabric (surface modified, coated, or nanofiller layered on carbon fabric) and CFRP composites for aircraft applications. Further, due to their functional and structural properties, hybrid CFRP composites (carbon fibre/fabric used with nano/micro filler as reinforcement) have become more attractive. The key results of researchers who studied the EMI SE of engineered carbon fibre/fabric and hybrid CFRP composites are represented in Table 1.

The literature shows that hybrid composites consisting of carbon fibre/fabric as primary reinforcement and fillers as secondary reinforcement are attractive replacements for metal electromagnetic shielding materials. The EMI SE in hybrid composites depends on factors like (i) Filler dispersion and concentration: The judicious quantity of filler leads to uniform dispersion and freedom from agglomeration. Because of this, stress concentration was reduced, and the mechanical and EMI SE of the composites were improved. (ii) Filler type, shape, size, and combination: The type of filler (such as carbon nanotubes or graphene nanoplatelets, etc.), its morphology, and its proportion in the composite significantly affect EMI SE^9–11,24^. Recently, graphene nanoplatelets (GNPs) have been incorporated into composites due to their remarkable properties, including (i) a large surface area: GNPs provide more interaction sites for strengthening the composite material. (ii) Excellent thermal conductivit**y**: GNPs help dissipate heat, improving the thermal stability of the composite. (iii) High Young’s modulus and electron mobility: GNPs enhance mechanical properties and electrical conductivity^12,17,18,20,21^. In the current research, GNPs are being utilized as a secondary reinforcement to epoxy resin and BD carbon fabric as primary reinforcement to further enhance the structural and EMI SE of resultant hybrid composites.

**Table 1:** Material combination and EMI SE of engineered carbon fabric and carbon fabric composites.

Primary reinforcement		Secondary reinforcement		Matrix	t(mm)	EMISE			Ref
Type of fabric/fibre	Area density (g.m^−^²)	Type of filler	Concentration (Wt.%)			SE_A_	SE_*R*_	SE_T_	
Plain Weave/Plain-3 K-T300	250	Nickel	1	Bisphenol A type Epoxy	1	23	12	35	^3^
3D carbon fabric mat/ACG-3 K200 T-1000	200	Ni & Ni Fe_2_O_4_	10–40	ABS	0.92	10	52	62	^4^
Carbon fabric/WOS1002	125	Ag nanowires	0.78	PU	< 0.5	10	100	110	^5^
Continuous carbon fabrics	147	Ag Nanoparticle ink	25	−	460 μm	101	14	104	^6^
UD Fabric Plain weave/T300-3 KCF	213	Conductive Silver complex	91.46%		10	−	−	52	^7^
Continuous carbon fiber	−	−	66%-fibre const.	Low-melting polyaryletherketone	0.38(6- layers)	40	20	60	^8^
PAN-based carbon fibre/T300-3 K CF	240	PAI/Ti_3_C_2_Tx nanosheets	4	PEEK	2	33	11	42	^9^
PET Nonwoven fabric	150	SWCNT/rGO	2	Di glycidyl, either of bisphenol A Epoxy	0.6	38	2	40	^10^
Glass Fabric	340	MWCNT/SCF Fe_3_O_4_ Nano particles	1-Fe_3_O_4_ 2000 SCF/mm^2^ 0.3-MWCNT	Epoxy	10 Layers	82	4	86	^11^
Plain woven CF	200	MWCNT Continuous GO filaments	15 MWCNT	Epoxy	1	−	−	32	^12^
UD carbon fabric/T700SC-12 K-60E, Qusi-isotropic [0^°^/90°/45°/−45°]4 s	300		−	Polyamide − 6	2	46	13	59	^13^
Continuous GF woven mat		TiO_2_ and Co nanoparticles	1	Bisphenol A type Epoxy	−	−	−	101	^14^
UD-CF	180		10 layers	Epoxy	2.3	18	9	27	^15^
Pitch-based XN80 Continuous Carbon Fiber pre-pregs $\:\left(\right[/\:{90}^{0}/\:{0}^{0}/\:{90}^{0}$]	125		4 layers	Epoxy	0.55	16	10	26	^16^
Porous CF mat with	200	Ag/PDA	30 Layers		30	72	30	102	^17^
Continuous carbon fibre fabric	300	MWCNT	3	Epoxy	2	40	11	51	^18^
UD prepreg/CCF800H/AC631	135	Carbon nanofibre sheet	10	C-P-P-C	2.2	18	6	24	^19^

## Materials

The bi-directional (BD) oriented plain weave carbon fabric (material batch No. W21 A31/3298). supplied by M/s. Bohr Chemical & Plastics Pvt. Ltd., Nasik, India, is used as primary reinforcement. The area density of the carbon fabric and the type of fibre were $\:160\:\text{g}.{\text{m}}^{-2}\pm\:5\text{\%}$ and 3 K, respectively. Bisphenol-A epoxy resin is used as a matrix with a suitable hardener. Graphene Nanoplatelets procured from M/S Ad-nano. The details of Graphene (CAS NO.:7782-42-5) have purity-99%, thickness-5 to 10 nm, surface area~110 m^2^. g^−1^, bulk density-0.166 g.cm^−3^ used as secondary reinforcement.

## Methods

A variety of solvents, such as Tetrahydrofuran (THF), Dimethylformamide (DMF), acetone, ethanol, Dichloromethane (DCM), Methyl ethyl ketone (MEK), water, etc., have been used for graphene dispersion by various researchers. For this study, laboratory-grade ethanol was used as the solvent. Graphene dispersion was achieved via ultrasonication and mechanical stirring to ensure homogeneity. Further, the carbon fabric-reinforced neat epoxy (CF/E) composite, GNPs and carbon fabric-reinforced epoxy (CF/GNP/E) composite laminates were prepared by hand layup. According to test standards, the specimens were cut by an abrasive waterjet machine to investigate mechanical properties and EMI SE. The Matsuzawa microhardness testing machine measured Vickers’ hardness (VH) (Model No. MMT-X7 A, Japan). Tensile strength, Young’s modulus and strain to failure were investigated for all the composite samples by testing as per ASTM D 638 − 10 and plotting the stress-strain curve. Five samples were analysed in each type of laminate, and an average reading was taken. The surface morphology of the GNPs, fractured surface morphology of the test specimen, and filler dispersion in the composite laminates were investigated by scanning electron microscopy (SEM; CARL, ZEISS, Germany). The EM shielding by absorption, reflection, and EMI SE was computed using S parameters, which were measured using a network analyser.

## Results and discussions

### Morphology of carbon fabric and GNPs

**Fig. 1:**
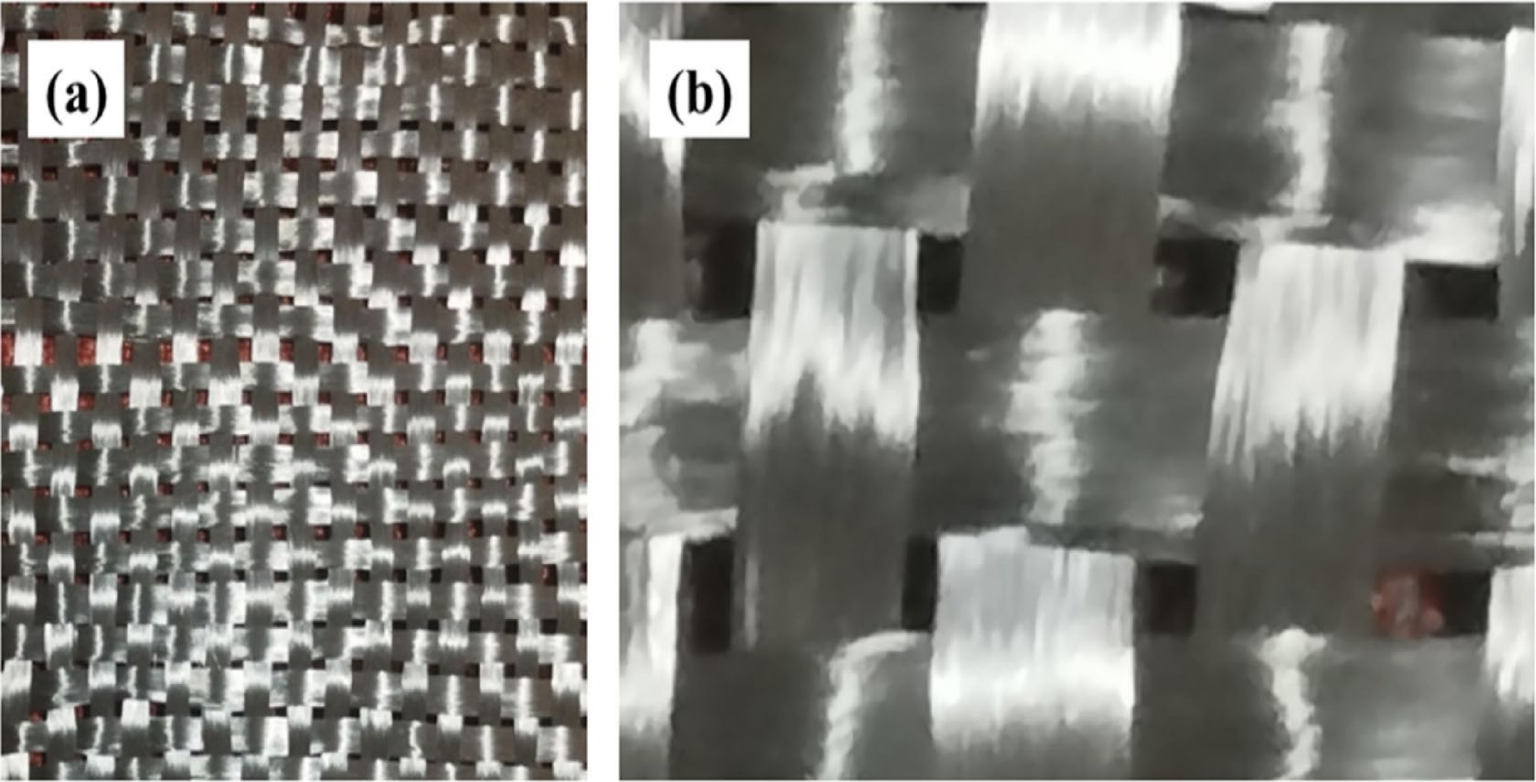
Microscopic view of bidirectional plain fibre fabric.

Figures 1(a) and (b) show magnified images of the carbon fabric used in this study. It is confirmed that the carbon fabric of BD is a plain weave type and free from damage.

**Fig. 2:**
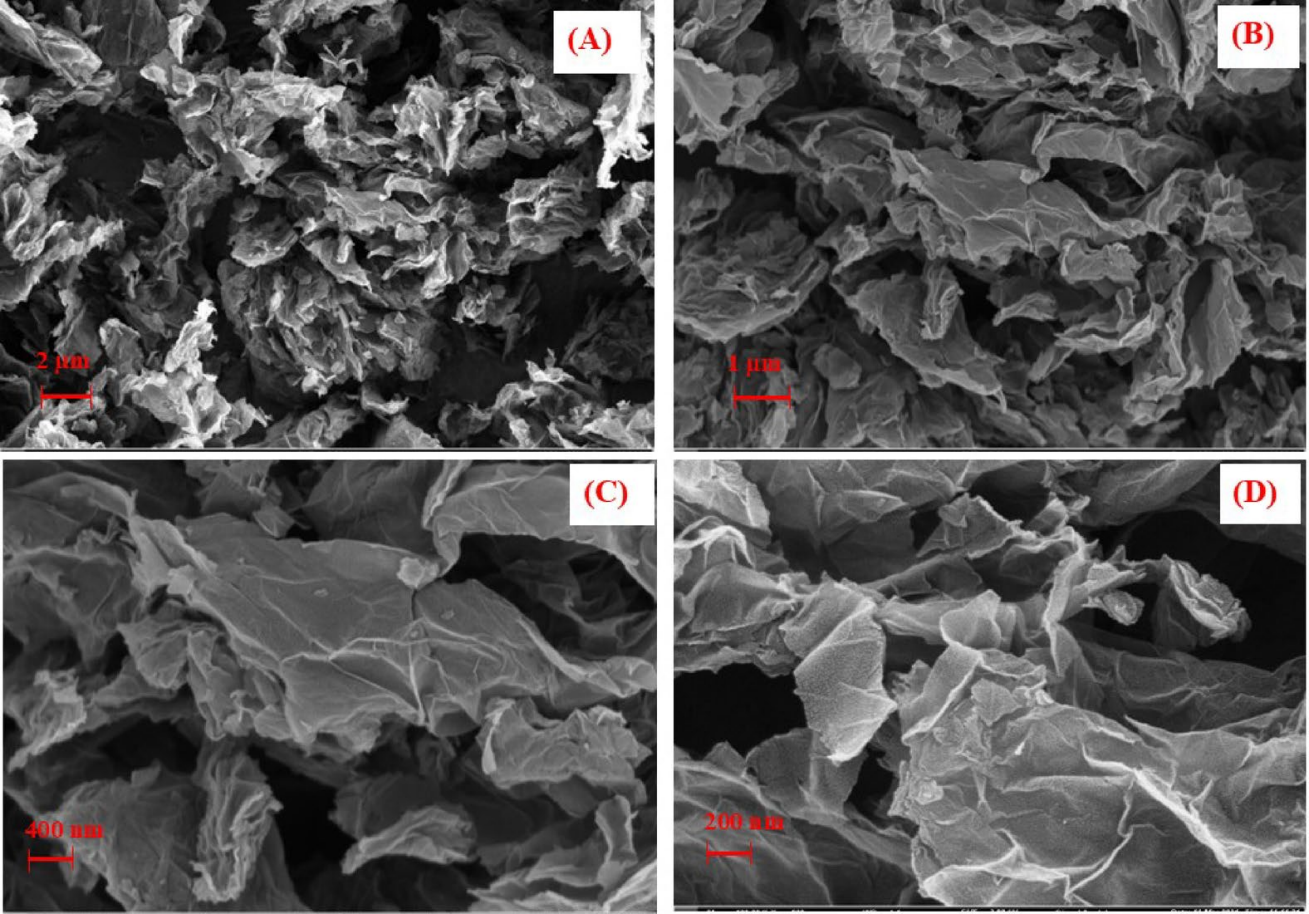
FESEM images of GNPs at various magnification (**A**) 10k, (**B**) 25k, (**C**) 50k and (**D**)100k.

The surface morphology of GNPs was investigated via SEM (scanning electron microscopy) (SEM; CARL, ZEISS, Germany) analysis. The SEM images of GNPs at various magnifications were taken and presented, as shown in Fig. 2. Figure 2(a) shows the SEM image of GNPs at 10 K magnification. The GNPs exhibited a lateral 2D flake structure with staggered layers. Each staggered layer consists of a few layers of GNPs, forming a multi-layer structure. Further, by investing at 25k, 50k magnification is presented in Fig. 2(b-c). The SEM images at these magnifications provide a clearer view of the lateral structure of the GNP flakes. The flakes have varied lateral sizes. To understand the thickness and surface morphology of GNPs, SEM images at 100k magnification are presented in Fig. 2(d). At this high magnification, the thickness of each GNP is revealed to be in the range of 5 to 8 nm.

### Structure-property of GNPs

**Fig. 3:**
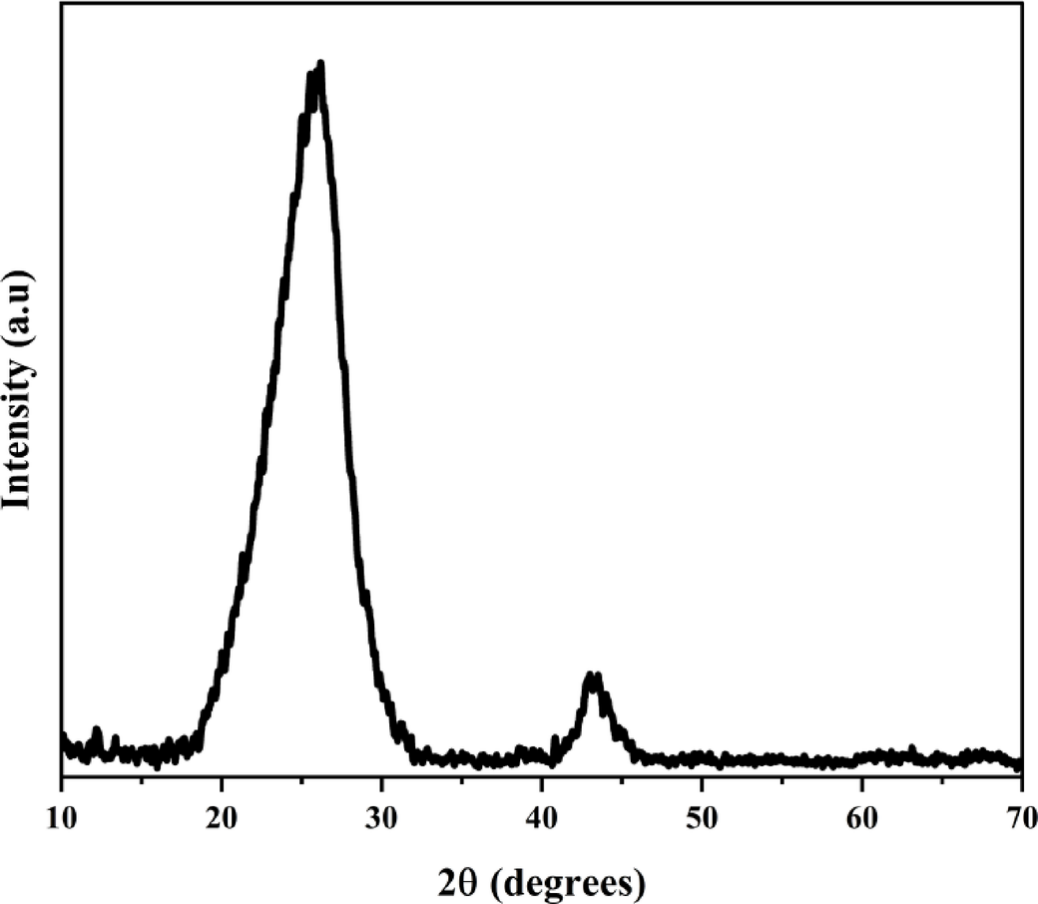
X-ray diffraction (XRD) pattern of GNP.

The crystalline structure of the GNPs was investigated using powder X-ray diffraction (XRD, Rigaku MiniFlex 600, Japan) from 20º to 70º at a speed of $\:{0.5}^{0}{m}^{-1}\:.$ Figure 3 shows the X-ray diffraction pattern for GNPs. It is observed that the position of the XRD peak falls at the value of$\:\:2\theta\:$ 25.4º confirmed GNPs. The average crystallite size (D_XRD_) of GNPs was calculated from Scherrer’s Eq. (1), and the $\:{d}_{002}$ (d-spacing for 2 H (002) from $\:2\theta\:$ peak at 25.4º) was calculated according to Bragg’s Law using Eq. (2).

1 $$\:{D}_{XRD}=\frac{k\lambda\:}{\beta\:cos\theta\:}$$

2 $$\:{d}_{002}=\frac{n\lambda\:}{2sin\theta\:}$$

where θ is the Bragg diffraction angle, $\:\beta\:$ is the full width at half maximum (FWHM), $\:\lambda\:$ Is the wavelength of the Cu-K $\:\alpha\:$ radiation (0.15406 nm), and $\:k$ is the shape factor (0.9). From the $\:{X}_{RD}$ spectra using Scherrer’s formula, the computed crystallite size (D_XRD_) is 2.26 nm and d spacing ($\:{d}_{002}$) is 3.5 Å. The physical origins of the broad (002), such as reflections, were interpreted from the uniform interlayer spacing (d_002_). The numbers of GNP layers (Nc) were calculated by using the interlayer spacing (d_002_) and apparent crystallite size (D_XRD_) in the c-c-direction by the Eq. (3), and it is found that 6.47, which is approximately 6, is confirmed by the supplier data.

3 $$\:{N}_{C}=\frac{{D}_{XRD}}{{d}_{002}}$$

### Matrix preparation

The dispersion of GNPs into epoxy is a crucial process while preparing CF/GNP/E composites. A good dispersion of GNPs with epoxy ensures a maximum reinforced surface area, which increases mechanical properties and affects the polymer chain internally, matrix strength and other functional properties. However, the high viscosity of epoxy hinders the GNP dispersion. Hence, ethanol is used as a solvent to improve the dispersion of GNPs in epoxy in the present work^22^.

**Fig. 4:**
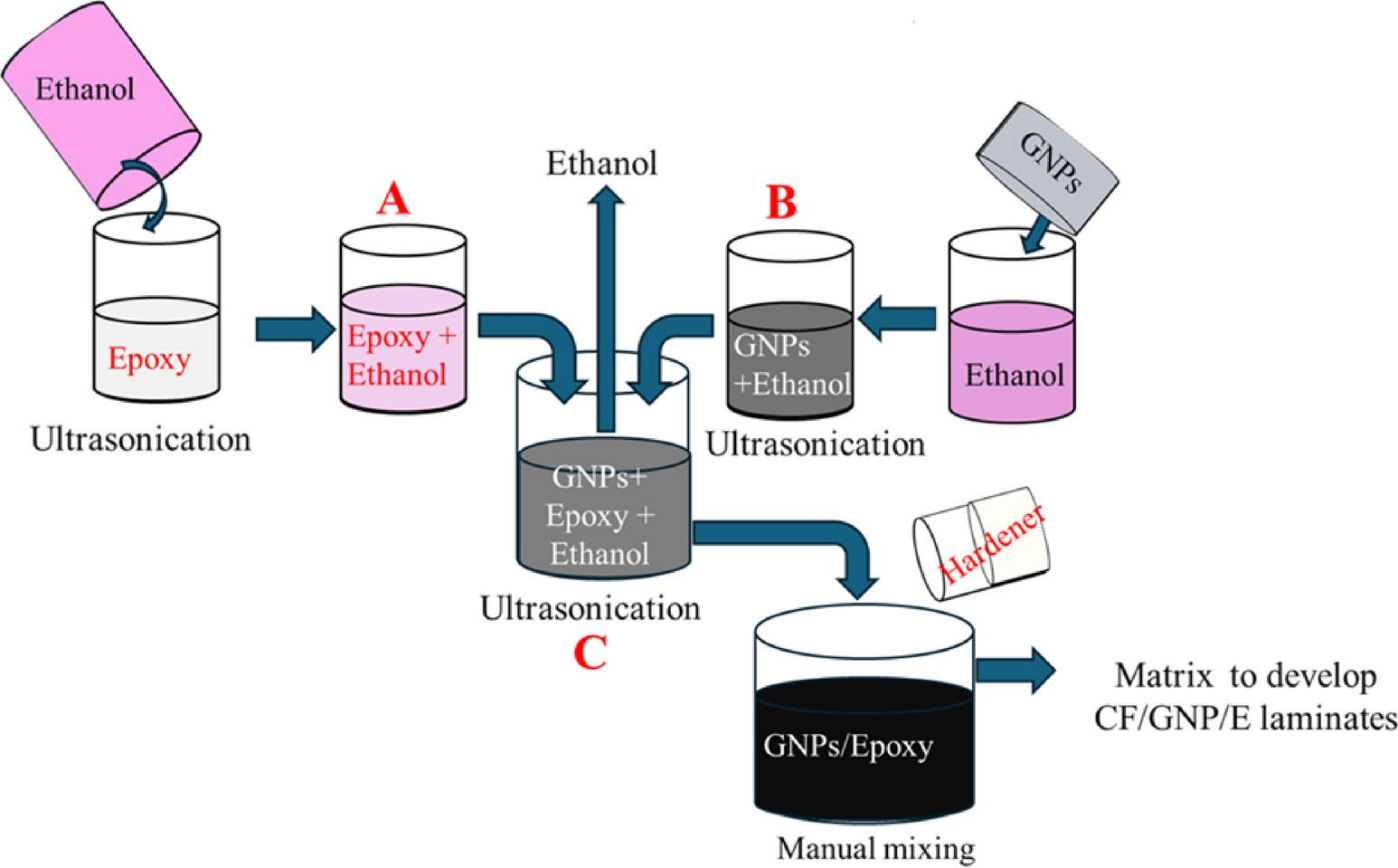
Preparation of Graphene-filled epoxy matrix.

Figure 4 shows the steps followed for preparing the GNP/E epoxy matrix. Initially, an equal volume of ethanol and epoxy was measured and mixed with a magnetic stirrer for about 30 min at 800 rpm, and the resultant solution was called solution (A) Simultaneously, GNPs were dispersed in ethanol by probe-type ultrasonication in a separate beaker. The ultrasonication was performed at a frequency of 20 kHz for about 30 min to ensure effective deagglomeration of GNP. The resultant solution is called as solution (B) Further, solution B was mixed with solution A by ultrasonication for about 2 h at 20 kHz. The resultant solution was called solution (C) Finally, solution C was mixed with hardener at a ratio of 100:80 by mechanical stirrer for about 5 min at 300 rpm and used as the matrix for carbon fabric and GNPs filler reinforced epoxy (CF/GNP/E) composite laminates preparation. The above method of matrix preparation was repeated by varying the quantity of GNP filler from 1 to 3 wt% in steps of 1 wt%.

### Preparation of CF/E and CF/GNP/E composite laminates

As supplied, the BD plain weave type carbon fabric was cut into 100 mm × 100 mm and immersed in the acetone to remove sizing agents, dirt, impurities, amorphous carbon, grease, etc. from the fabric. After cleaning with acetone, the fabric was immersed in deionised water and kept for 10 min in a hot air oven to remove water and moisture. In the present study, the carbon fiber fabric is impregnated with epoxy resin (with or without GNP) using hand layup methods. Further, the layers are consolidated one above the other using a roller and brush, and air bubbles are removed by vacuum. A releasing film was kept on the glass plate before placing the carbon fabric and after completing the layup to remove the laminate^27^easily. The laminates are then cured under compression mode at room temperature for around 24 h. Post-cure at 150–180 °C for further crosslinking for about 2 h. After complete curing, the laminates were taken out of the glass mould. The process was repeated to develop 1-layer, 2-layer and 4-layer CF/1GNPS/E, CF/2GNP/E and CF/3GNP/E composite laminates^28^. Table 2shows the material composition and designation of composite laminates. According to ASTM standards, the test samples were prepared by a computerised CAD-operated abrasive water jet machine from the prepared laminates. Optimized filler concentration in the composites gives better performance and improves tensile and flexural properties due to enhanced reinforcement, uniform load distribution, and transfer of load from matrix to filler and fiber. However, higher concentrations of fillers led to stress concentration spots in the composite due to improper resin impregnation, improper wetting and poor interface between filler, fiber and matrix. From the literature, we noticed that the 1–3 wt% GNP reinforcement in epoxy matrix gives better mechanical properties^29^. Hence, in the present work, we selected 1–3 wt% GNP as filler concentration.

**Table 2:** Material composition and designation of CF/E and CF/GNP/E composites.

SL. No.	Type of composite	Sample designation	GNPs (wt%)	CF layers (No.)
1	Neat CF/E Composite	1 CF/E	Nil	1
2		2 CF/E		2
3		4 CF/E		4
4	CF/GNP/E composites	1 CF/1GNP/E	1	1
5		2 CF/1GNP/E		2
6		4 CF/1GNP/E		4
7		1 CF/2GNP/E	2	1
8		2 CF/2GNP/E		2
9		4 CF/2GNP/E		4
10		1 CF/3GNP/E	3	1
11		2 CF/3GNP/E		2
12		4 CF/3GNP/E		4

### FTIR spectral analysis

**Fig. 5:**
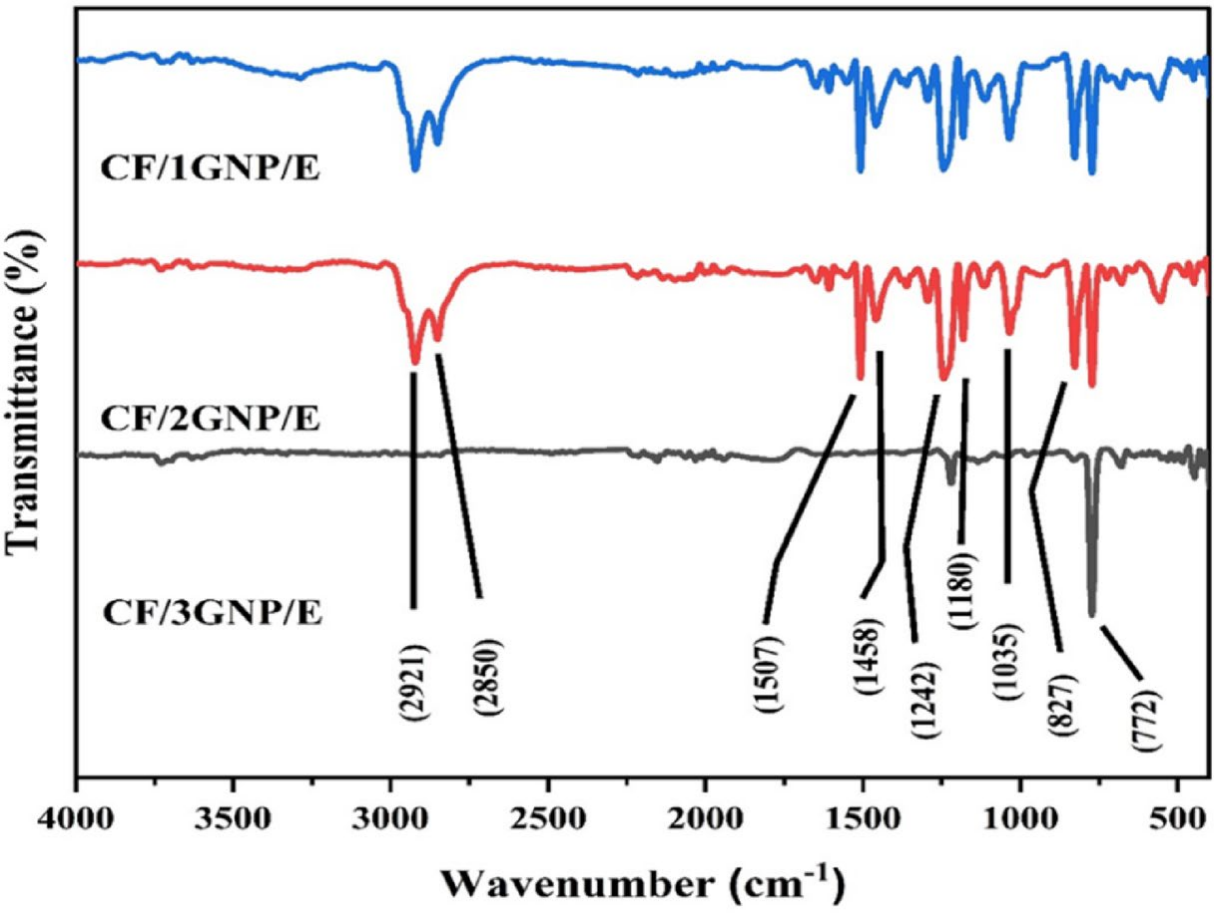
FTIR Spectra of CF/GNP/E composites.

The Fourier transform infrared (FTIR) spectral analysis was performed on the CF/GNP/E nanocomposites of different GNP compositions to identify the functional groups and investigate the interaction between the GNP and CF-reinforced epoxy matrix and the FTIR spectra, as shown in Fig. 5.

The FTIR spectrum of the CF/GNP/E composite matrix shows significant peaks at the frequencies 2921, 2850, 1608, 1507, 1458, 1458 1458 1458,1242, 1180, 1035, 827, and 772 cm^−1^. The peaks at 2921 cm^−1^ and 2850 cm^−1^ correspond to the –CH stretching vibrations of the epoxy ring and the symmetric stretching –CH vibrations of the –CH_2_ group of aromatic rings, respectively. The prominent peaks at 1608 cm^−1^, 1507 cm^−1^, and 1458 cm^−1^ may be due to the C–C stretching vibration of aromatic rings of neat epoxy. The signals at 1242 cm^−1^, 1180 cm^−1^, and 1035 cm^−1^ are possibly due to asymmetrical aromatic and aliphatic C–O stretching of the epoxy resin, whereas the signal at 827 cm^−1^ is assignable to –CH out-of-plane deformation in aromatic and epoxide ring vibrations. The prominent absorption peak at 772 cm^−1^represents the bending vibrations of the epoxy functional group in the resin. These signals resemble the vibrations of epoxy resin reported in the literature^23^. Additionally, the feeble peak around 3400 cm^−1^ represents the O-H bond stretching vibrations in the epoxy. The minor peaks observed at 1650 cm^−1^ indicate C = O stretching vibration from the carboxylic group of GNPs. The carbon fibre in the composite is not expected to alter its vibrational characteristics as it lacks major interacting groups. Therefore, the dominant interaction is observed between the GNP and epoxy resin in the composites. The spectrum of CF/GNP/E for different filler compositions of GNP reveals micro-level changes in the spectrum as the GNP concentration is increased from 1 to 3%. The noticeable changes were observed in the intensity and position of peaks in the frequency range of 1000 to 3000 cm^−1^. The enhancement in bending vibrations of the epoxy functional group of the resin (772 cm^−1^) was dominant. The weakening of O-H, C-H, and C-O bond stretching vibrations of epoxy resin and the C = O bond stretching vibration of GNPs is evident from the spectral data. This indicates substantial interaction between the C = O group of GNPs and the functional groups of epoxy resin. The shifting of leading bands and variation in the intensity of peaks suggest the successful formation of composites through considerable physical or Van der Waals interactions between epoxy and GNPs, resulting in an overall reinforcing effect on the composite.

### Mechanical properties of CF/E and CF/GNP/E composites

#### Hardness study

Vickers hardness (HV) measures the material’s resistance to indentation and is commonly used to characterise the hardness of metals, polymers, and composite materials. Vickers’ CF/E and CF/GNP/E composites’ hardness is studied using a Matsuzawa microhardness testing machine (Model No. MMT-X7 A, Japan). It is computed by applying a load onto the composite sample through a pyramid-shaped indenter and measuring the average diagonal length of the indentation left in the material. A load of 1000 g is applied for 20 s, and the Vickers hardness (HV) in N/mm² is calculated using Eq. (4).

4 $$\text{HV }=1.891\frac{\text{F}}{{\text{d}}^{2}\:}$$

Where F is the load applied on the diamond point indenter (N), and d is the average of the diagonal of indentation developed by diamond tip indentation on the specimen (mm^2^). In each sample, 10 readings were taken, out of which five readings of very near values are reported in the table; finally, those with very little difference were considered to calculate the average value and tabulated in Table 3. The HV depends on the type of fabric, matrix, quantity of filler, and manufacturing type of the composites. In the present study, only the filler concentration was varied from 0 to 3 wt%, which is not a significant quantity as per the literature concerned with harness studies of carbon fabric-reinforced filler-loaded composites. Hence, consistency was observed in the average HV values in all the composites.

**Table 3:** Vickers’ hardness of CF/E and CF/GNP/E composites.

Sample	Trial-1	Trail-2	Trail-3	Trail-4	Trial-5	HV _Aver_.
CF/E	42.5	46.5	40.7	38.4	41.4	41.9
CF/1GNP/E	44.3	43.8	41.8	41.3	33.8	41
CF/2GNP/E	37.2	39.1	44.7	46.3	40.2	41.5
CF/3GNP/E	40.7	34.6	37.5	39.2	39	38.2

#### Tensile behaviour of CF/E and CF/GNP/E composites

ASTM D638 is the most common testing standard for determining the tensile properties of reinforced composites^24^. Tensile test was conducted for CF/E and CF/GNP/E composite samples as per ASTM D638. Sample type-5 of dumbbell shape with sample dimensions such as the overall length: 63.50 mm, gauge length: 9.53 mm, width: 3.20 mm, thickness: 3.18 mm, grip length: 9.53 mm was used. The crosshead speed of 5 mm/min was maintained for all the samples. The tensile test data drew the stress-strain curve, and tensile strength, Young’s modulus, and strain-to-failure were estimated.

**Fig. 6:**
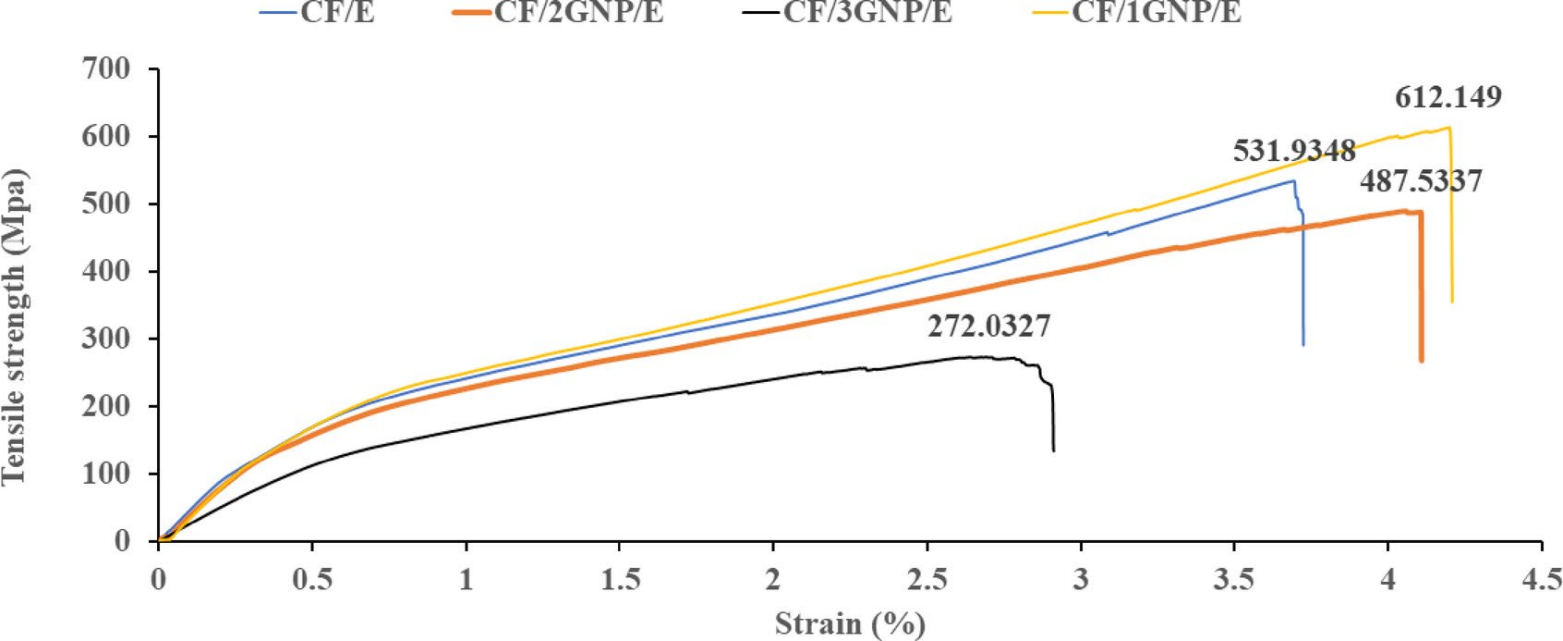
Stress-Strain Curve of CF/E and CF/GNP/E Composites.

**Fig. 7:**
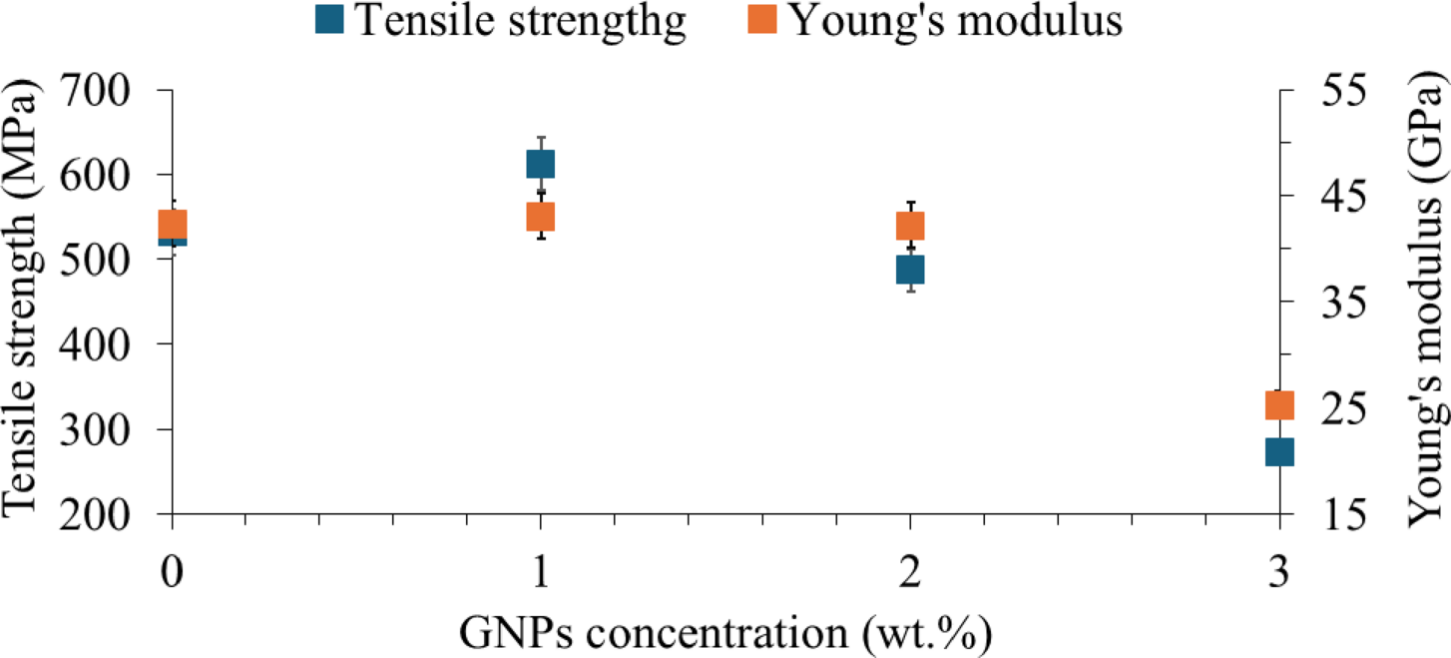
Tensile strength and Young’s modulus of CF/E and CF/GNP/E composites.

The tensile behaviour of CF/E composite and CF/1GNP/E. CF/2GNP/E and CF/3GNP/E are shown in Fig. 6. It was found that adding GNPs to the epoxy matrix alters tensile behaviour. The CF/1GNP/E showed significantly improved tensile strength and strain-to-failure compared to CF/E. This is attributed to the large surface area of GNPs, which leads to enhanced interfacial bonding between the matrix and reinforcement, which improves the reinforcing effect and better load transfer from the matrix to the reinforcements. Also, uniformly dispersed GNPs in the epoxy matrix increased the toughness and ductility of the resultant composites. Further, it is noticed that strain-to-failure is improved due to the addition of 2 wt% GNPs, but the tensile strength and modulus were decreased. This is because, at 2 wt% filler, filler-loaded epoxy becomes more ductile than neat epoxy. Also, the GNPs enter the molecular chain of epoxy, which restricts its cross-linking ability and reduces strength (refer to Fig. 6) and stiffness, as mentioned in Fig. 7. From the tensile behaviour of CF/E, CF/1GNP/E and CF/2GNP/E, it was found that the GNPs in the epoxy matrix changed the mode of failure from brittle to ductile behaviour. From the literature, it is found that the improvement of tensile strength and strain-to-failure of carbon fabric and GNP-reinforced epoxy composites at lower GNP concentrations (< 2wt.%)^22^. Similar results were obtained in the present research study, which further added 3 wt% GNPs to epoxy leads to agglomeration of GNPs and poor wetting of the matrix with reinforcement. This leads to stress concentration, and void formation leads to early failure, as shown in the stress-strain plot^30,31^. From the above discussion, the tensile properties point of view shows that the optimum concentration is one wt% GNPs are beneficial. The elongation of the tensile specimen at the break is as shown in Fig. 6. It was found that the CF/1GNP/E and CF/2GNP/E samples showed higher elongation before fracture than the CF/E samples. This indicates that the CF/E composites become more ductile due to the addition of GNPS. This is attributed to the flake structure of the GNPs in the matrix. However, the CF/3GNP/E composite sample showed more brittleness due to the higher concentration of GNPs in the matrix.

**Fig. 8:**
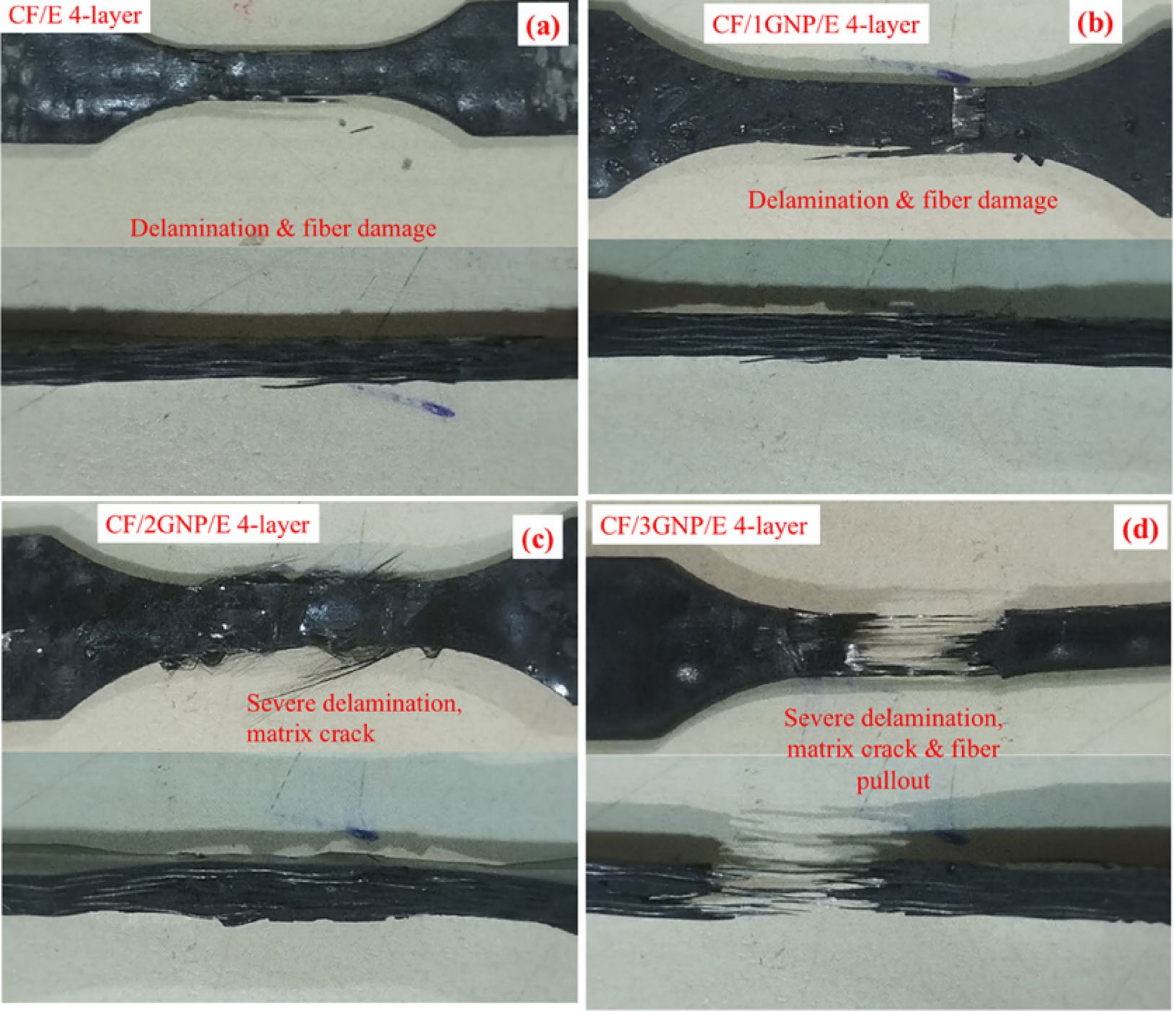
Fractured Tensile Specimens of CF/E and CF/GNP/E Composites.

To understand the failure mechanism, the tensile failure samples are magnified, investigated, and presented in the digital photographs in Fig. 8(a-d). It is observed that the failure of the tensile specimen was due to the delamination of fabric layers and fibre damage of the CF/E and CF/1GNP/E composites. Both fractured samples in Figs. 8(a) & 8(b) reveal the purely ductile failure of the fabric in the composites. Also, higher slippage of layers was observed in CF/1GNP/E composite compared to CF/E; this is evidence for higher tensile strength and strain-to-failure observed in the stress-strain plot. It is attributed that the addition of 1 wt% GNP filler leads to good load transfer from matrix to fibre/filler. It demonstrates a better interface & enhanced load-bearing capacity before failure. As the filler concentration increases, the interface between reinforcements and resin will become poor, reducing the strength and modulus of the fracture surface of 2 wt% and 3wt.% GNPs loaded CF composites are shown in Fig. 8(c -d), and more delamination and interlaminar matrix failure were noticed due to poor interface. The 3 wt% filler loaded composites failed specimens reveal failure by delamination, severe matrix failure due to inferior interface strength. Both 2 wt% and 3 wt% filler-loaded composites showed the brittle failure of the matrix due to weaker load-carrying capacity and poor integrity of matrix, filler, and fibre, as shown in Fig. 8(d).

## Electromagnetic shielding effectiveness

The EMI SE of a material is a measure of its ability to block or attenuate electromagnetic waves that are incident on it. The shielding effectiveness (SE) of a material in this research is quantified by the coaxial transmission line method by measuring the S-parameters (scattering parameters: S11, S12, S21, S22) using a 2-port Keysight Technologies N19918 A MY58312077 vector network analyser with WR-90 waveguide holder. The S11 (Reflection coefficient at port 1) indicates how much power is reflected from the material at a given frequency. S21(Transmission coefficient from port 1 to port 2) indicates how much power passes through the material from port 1 to port 2. S12 and S22, in most cases, are less critical for shielding measurements. Hence, it was not taken into consideration. The key S-parameter for shielding effectiveness is S21, which measures how much energy passes through the material, directly related to the shielding effectiveness.

**Fig. 9:**
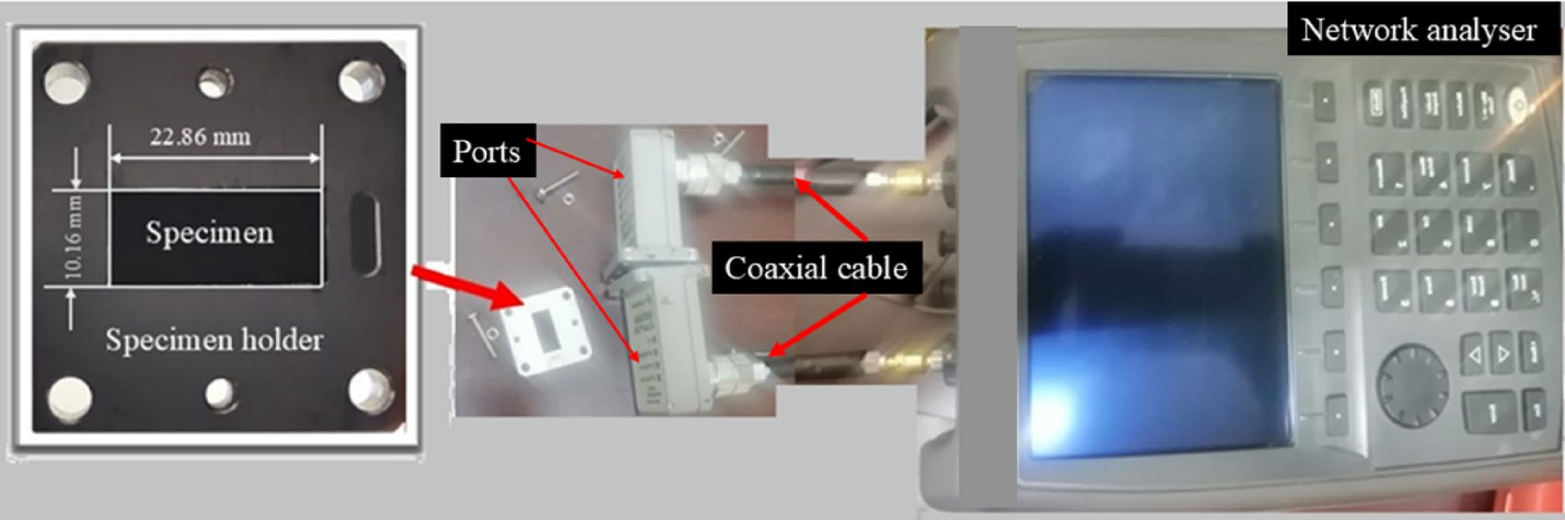
EMI SE measurement setup.

In this research, specimens of a dimension $\:\:22.86\:mm\times\:10.16\:mm$they were cut by CF/E and CF/GNP/E laminates using abrasive water jet machining. The prepared samples were placed on a WR-90 waveguide sample holder and scattering parameters (S-parameters) were measured over a frequency range of 8 GHz to 12 GHz for all the samples. Before placing the sample, an S-parameter measurement and a full two-port calibration were carried out. The experimental setup used for EMI SE is given in Fig. 9.

Further, the shielding effectiveness by absorption ($\:{SE}_{A})$ and reflection ($\:{SE}_{R}$) is calculated using S parameters through Eqs. (5) and (6), respectively.

5 $$\:{SE}_{A}\:\left(\text{d}\text{B}\right)\:=\:10{log}_{10}\left[\frac{1-{{S}_{11}}^{2}}{{{S}_{21}}^{2}}\right]$$

6 $$\:{SE}_{R}\:\left(\text{d}\text{B}\right)\:=\:10{log}_{10}\left[\frac{1}{1-{{S}_{11}}^{2}}\right]$$

The total shielding effectiveness ($\:{SE}_{T}$) was calculated using the Eq. (7)

7 $$\:{SE}_{T}=\:{SE}_{A}+{SE}_{R}$$

**Fig. 10:**
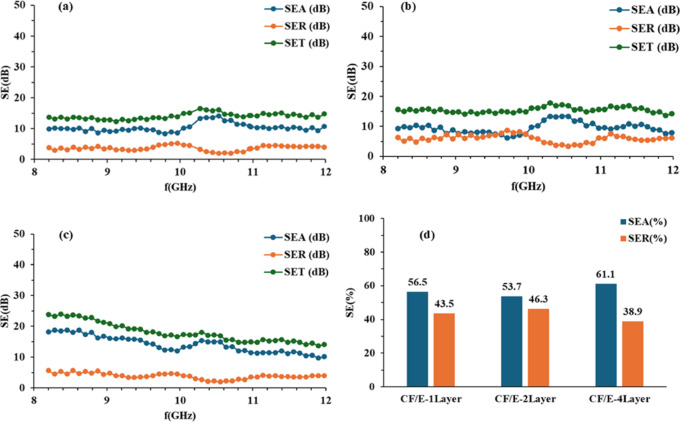
SE_A_, SE_R_ and SE_T_ of (**a**) CF/E-1Layer; (**b**) CF/E-2Layers (**c**) CF/E- 4Layers & (**d**) SE_A_ and SE_R_ percentage of all CF/E composites.

**Fig. 11:**
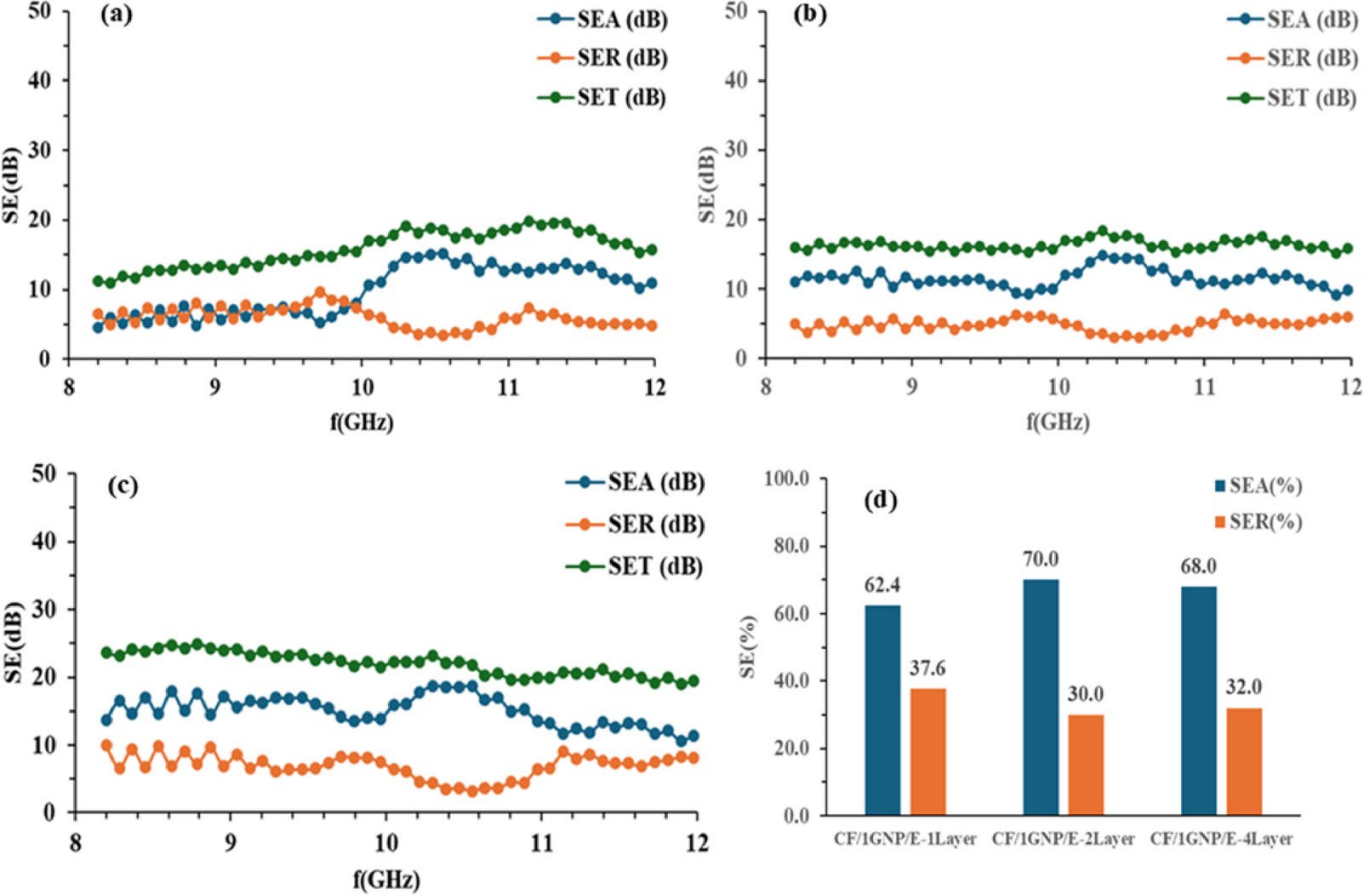
SE_A_, SE_R_ and SE_T_ of (**a**) CF/1GNP/E-1Layer; (**b**) CF/1GNP/E-2 Layers (**c**) CF/1GNP/E- 4 Layers & (**d**) SE_A_ and SE_R_ percentage of all CF/1GNP/E composites.

**Fig. 12:**
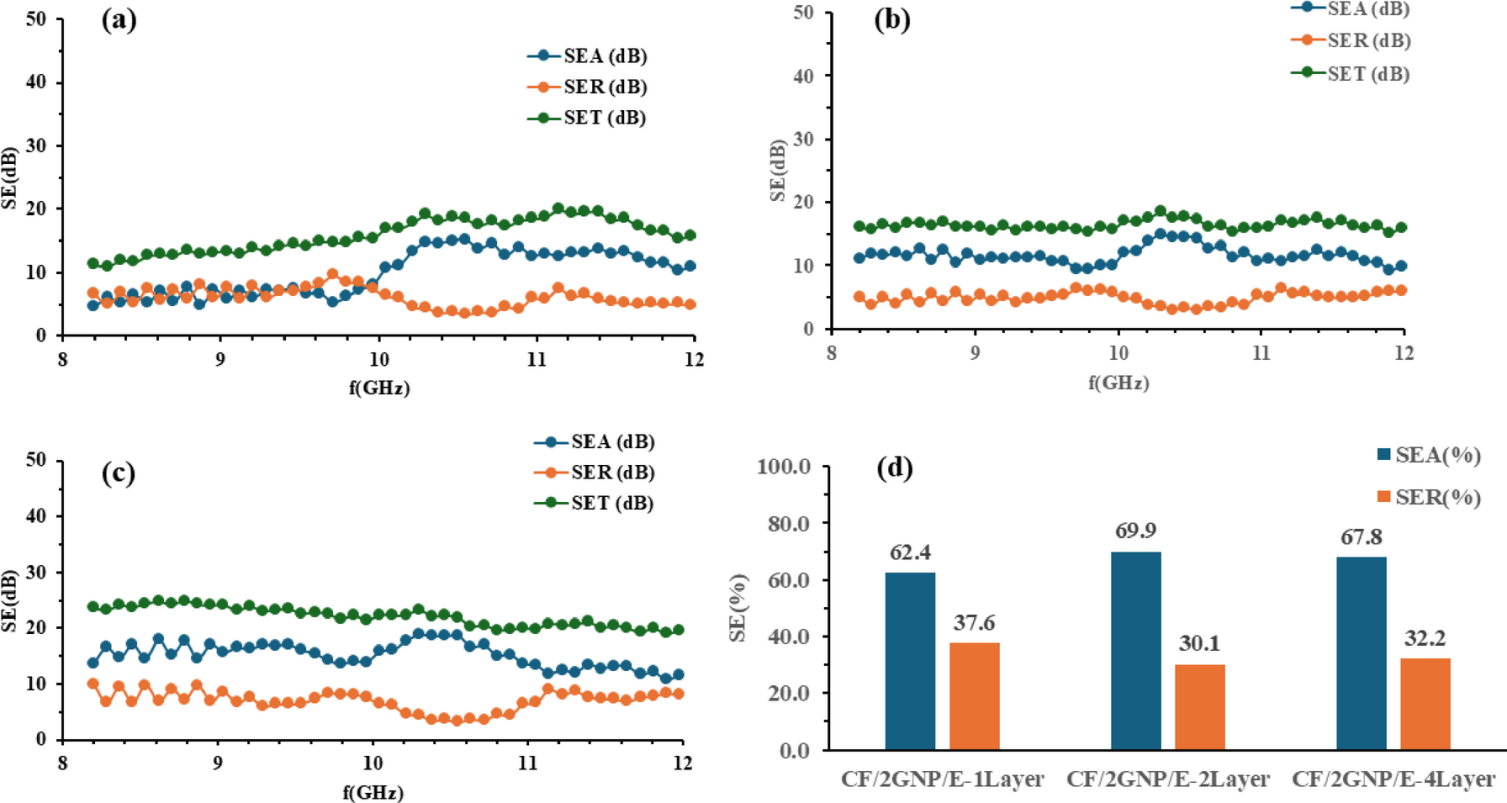
SE_A_, SE_R_ and SE_T_ of (**a**) CF/2GNP/E-1Layer; (**b**) CF/2GNP/E-2Layers (**c**) CF/2GNP/E- 4Layers & (**d**) SE_A_ and SE_R_ percentage of all CF/2GNP/E composites.

**Fig. 13:**
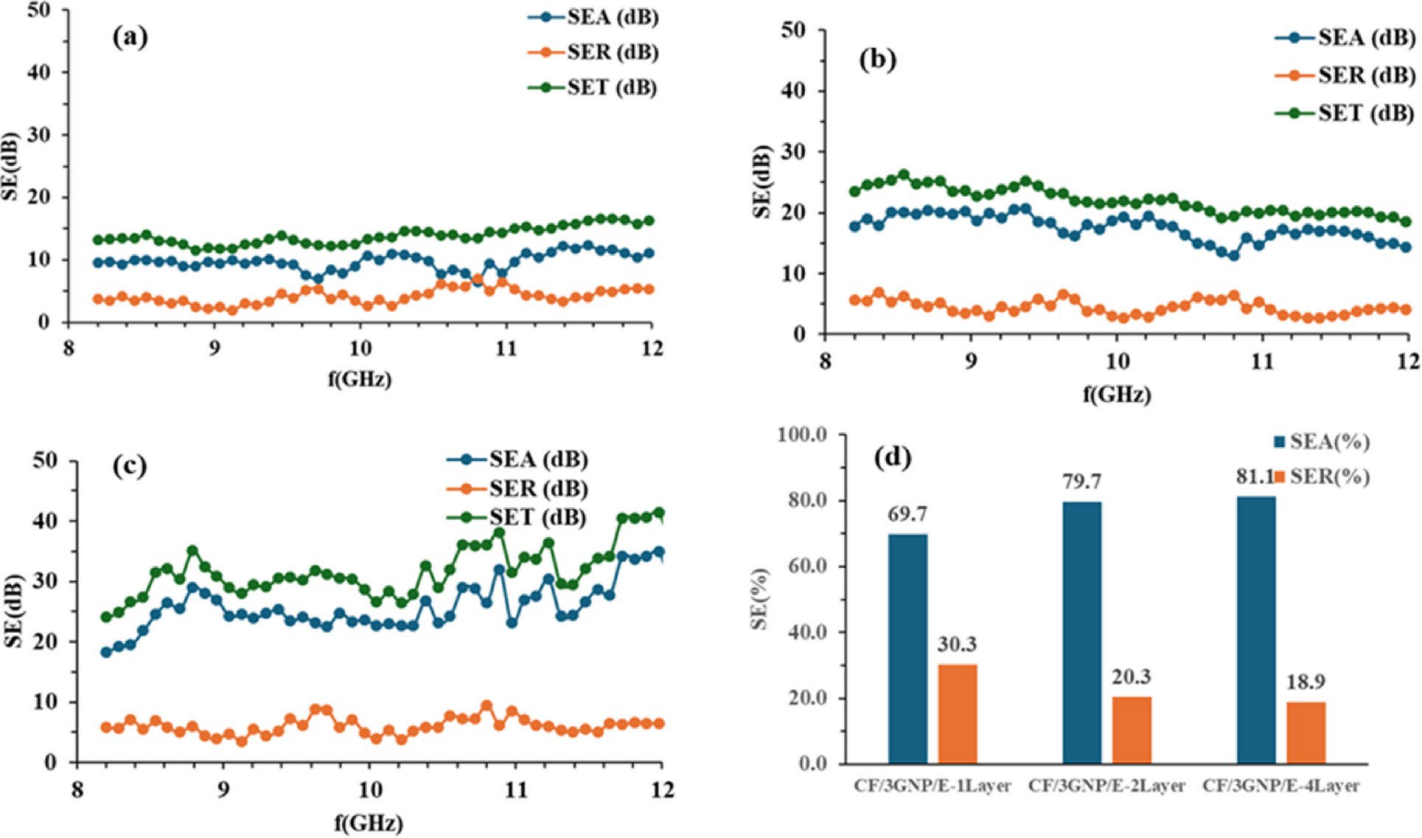
SE_A_, SE_R_ and SE_T_ of (**a**) CF/3GNP/E-1Layer; (**b**) CF/3GNP/E-2Layers (**c**) CF/3GNP/E- 4Layers & (**d**) SE_A_ and SE_R_ percentage of all CF/3GNP/E composites.

The $\:{SE}_{A},\:{SE}_{R}$ and $\:{SE}_{T}\:$of CF/E, CF/1GNP/E, CF/2GNP/E and CF/3GNP/E with one-layer, two-layer and four-layer samples at 8 to 12 GHz frequencies were plotted as shown in Figs. 10 (a-c), 11(a-c), 12 (a-c) and 13 (a-c), respectively.

Figure 10(a-c) shows the SE_A_ (Shielding effectiveness due to absorption), SE_R_ (Shielding effectiveness due to reflection), and SE_T_ (Total shielding effectiveness) of one-layer, two-layer and four-layer CF/E composite samples in the X-band range. It is observed that one-layer and two-layer CF/E composite samples exhibited SE_A_ independent of operating frequency in the selected X-band range. The four-layer CF/E composite showed frequency-dependent SE_A_. This is due to the skin effect. i.e. skin depth (δ) is inversely proportional to the square root of frequency(f), as shown in Eq. (8). Where δ is the skin depth, µ is the magnetic permeability (H/m), $\:\sigma\:$ is the material’s electrical conductivity (S/m), and f is the electromagnetic field frequency.

8 $$\:{\updelta\:}=\sqrt{\frac{1}{\pi\:\mu\:\sigma\:f}}$$

The δ decreases as the f increases, meaning that at higher frequencies, the EM waves travel on the surface of the composite and do not penetrate deeper into it. At low f, the δ is large, and the EM waves penetrate deeper into the composite and lead to more absorption. Hence, 4-layer performance is better at lower frequencies than at higher frequencies. Not much significant change in SE of one-layer and two-layer samples is observed. However, four-layer samples showed improved SE. This is due to the increased absorption and less reflection of the EM radiation because of the multiple layers of carbon fabric^26^. This is represented in Fig. 10(d). It was found that as the layers increased in the composites, the absorption-dominated shielding effectiveness increased. This is because the penetrated EM waves are attenuated in the multiple layers of carbon fabric.

Conventional metallic shields typically exhibit SE values of 60–100 dB, the proposed composite in this research work offers lighter weight, corrosion resistance, and a high strength-to-weight ratio. Hence, it is a more suitable alternative for applications where weight reduction is critical. Also, metals generally provide higher SE due to the reflection of EM waves. The reflected EM waves further develop interference. In this respect, proposed composites are absorption-dominated and reduce electromagnetic interference.

Figure 11(a-c) SE_A_, SE_R_, and SE_T_ of CF/1GNP/E composite of one-layer, two-layer and four-layer samples in the X-band frequency range. It is noticed SE_A_ increases from 62 to 70% in this composite due to the increase in one layer, as shown in Fig. 11(d). However, a similar trend was observed for SE_A_ and SE_R_ two- and four-layer composites. However, the SE_R_ of the CF/1GNP/E composite is less compared to the CF/E composite. This is due to the absorption of EM radiation by the GNP fillers. Hence, the addition of GNP makes the composite more absorption-dominated in SE.

Figure 12(a-c) SE_A_, SE_R_, and SE_T_ of CF/2GNP/E composite of one-layer, two-layer and four-layer samples in the X-band frequency range and Fig. 12(d) show the percentage SE. It is observed that the shielding effectiveness of both CF/IGNP/E and CF/2GNP/E composites showed similar results. It attributed no significant change in the conductivity of the composite by increasing GNP concentration from 1 wt% to 2 wt%.

Figure 13(a-c) SE_A_, SE_R_, and SE_T_ of CF/3GNP/E composite of one-layer, two-layer and four-layer samples in the X-band frequency range and Fig. 13(d) show the percentage SE. It was found that adding 3 wt% GNP in the composite increased the absorption shielding effectiveness due to an improved conductive network in the epoxy matrix. Further, it was also noticed that the absorption-dominating shielding increased due to the multi-layer effect in the composite. The CF/3GNP/E 4-layer composite showed the highest (32.4 dB) shielding effectiveness and was recommended for commercial application in the X-band frequency range.

**Fig. 14:**
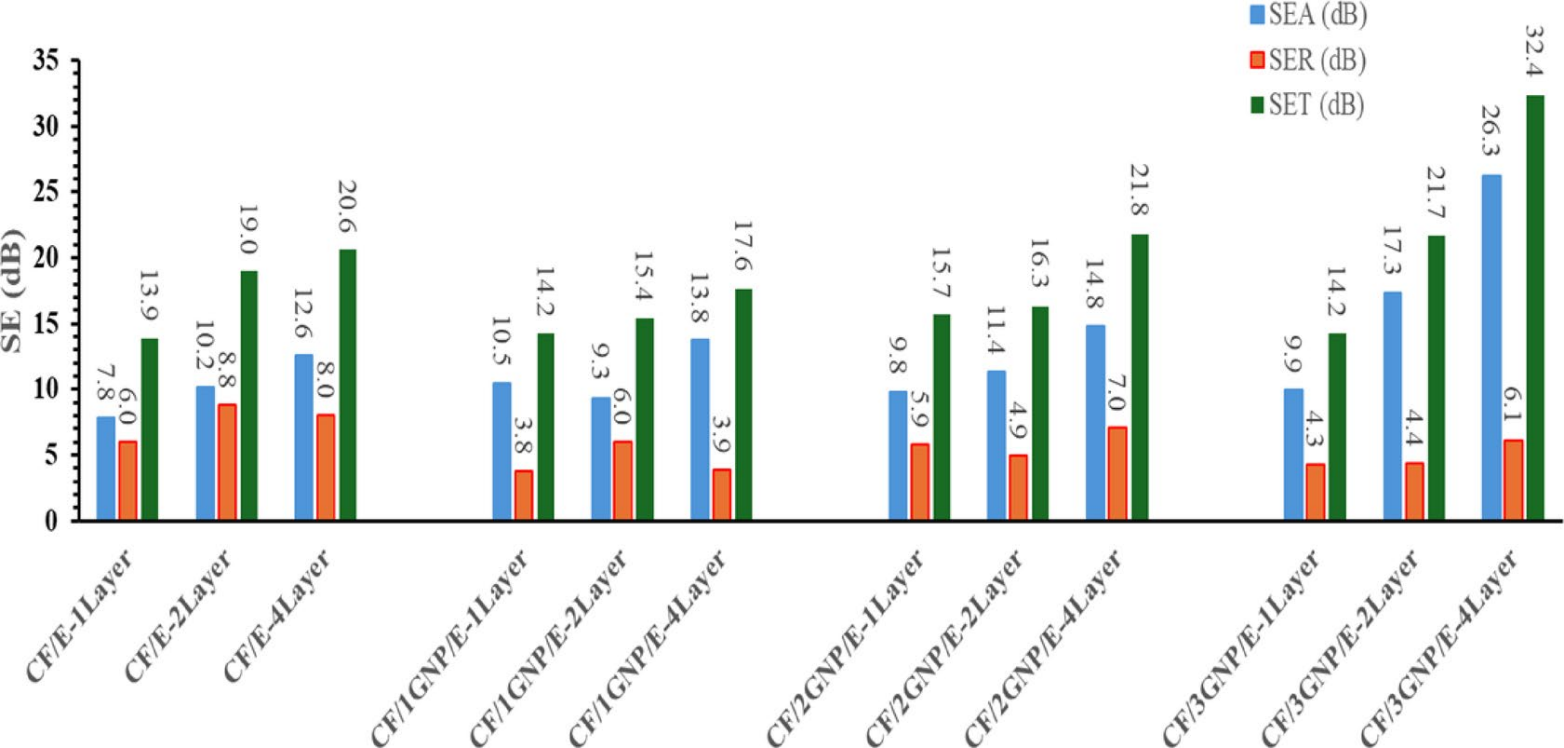
SE_A_, SE_R_ and SE_T_ of neat CF/E and CF/GNP/E composites.

Figure 14 shows the EMI SE overall comparison of CF/E composites with CF/GNP/E of one-layer, two-layer, and four-layer at the frequency range of 8 to 12 GHz. It was found that the shielding effectiveness by absorption increased as the layers increased in the composites. This is because the penetrated EM waves in the composite are attenuated in the two-dimensional plane of the carbon fabric. The shielding effectiveness by reflection was found to be less in 3 wt% filler-loaded composites because conductive GNP fillers develop network conductive paths and reduce shielding reflection.

The influence of defects in the composite samples plays a crucial role in determining both structural properties and EMI shielding effectiveness. For EMI shielding, surface defects may disrupt the conductive network formed by fillers like graphene nanoplatelets (GNP) or carbon fibers, reducing shielding effectiveness (SE). Voids or cracks can cause signal leakage, weakening the material’s ability to absorb or reflect electromagnetic waves. Agglomeration of conductive fillers may also lead to non-uniform shielding performance due to uneven distribution of shielding pathways. In the present research, the proposed composites are carefully manufactured, free from microcracks, and have reasonable voids (around 3 V%). Also, multiple samples were investigated for both mechanical and electromagnetic shielding properties. Hence, the results obtained are reasonable.

## Conclusion

In this work, the CF/E and CF/GNP/E laminates were successfully prepared using the hand layup method. Mechanical properties such as tensile strength, Young’s modulus, strain-to-failure, and hardness were investigated.

- It was found that 0 to 3 wt% GNP concentration is insignificant in hardness studies of carbon fabric-reinforced filler-loaded composites. However, a slight reduction in hardness was noticed due to adding 3 wt% GNP. This may be attributed to the void or agglomeration of GNP in the composites.
- The tensile strength, strain-to-failure, and stiffness of the CF/E composites improved due to the addition of 1wt.% GNP (Tensile strength: 612MPa > 531 MPa, Modulus: 43MPa > 42 MPa), but a higher concentration deteriorates the structural properties of the CF/E composite due to agglomeration.
- The EMI SE increased with the addition of GNPs to CF/E composites, and the multi-layer structure in the CF/E composites showed absorption-dominated electromagnetic shielding. The CF/3GNP/E 4-layer composite showed the highest (32.4 dB) shielding effectiveness and was recommended for commercial application in the X-band frequency range.

## Data Availability

The datasets generated during the current study are available from the corresponding author upon reasonable request.
